# Queen Charlotte's Lying-In Hospital

**Published:** 1919-10-25

**Authors:** 


### THE EDITOR'S LETTER-BOX.

[Correspondence on all subjects is invited, but we cannot in any way be responsible for the opinions expressed by our correspondents, who must give their name and address as a guarantee of good faith, but not necessarily for publication. Correspondents are reminded that brevity of style and conciseness of statement greatly facilitate early insertion.]

### QUEEN CHARLOTTE'S LYING-IN HOSPITAL.

### A Growing Demand for a Great Up-to-date Efficient Midwives' Training-School.

Reputation is the last thing to become established and the last thing to desert even ancient institutions. The continuance of an unblemished reputation and the continuity of the confidence of a great body of workers like the members who devote themselves to the practice of midwifery can be maintained only so long as everything affecting the efficiency of the training, comfort and just safeguarding of the health, adequate leisure and hours off duty is rigidly enforced throughout the training-school devoted to the training of pupil nurses, in the best interest of the pupil's welfare, happiness, and efficient equipment for her subsequent work as a midwife. Pupil nurses come to their work as novices to hospital life, its duties, the exact quality of much of the service required, and hygienic laws, or they would always obtain an adequate dietary with hot meals served in an appetising manner during the night. Recent experience proves in the history of institutions which have undertaken to train pupils, that some or all of these necessary safeguards and just enforcements have been allowed to lapse, or to be frequently absent from the conduct of such an establishment. In the case of Queen Charlotte's Hospital School for pupil nurses and others the issues of The Hospital of September 20, pages 619-20, and subsequently, contain a carefully prepared account of the abuses and evils which might attach to a pupil nurse who in all innocence may enter for training at Queen Charlotte's Lying-in Hospital.

Since our last issue we have had brought to our notice what is claimed to be the present position of affairs in regard to pupil nurses at Queen Charlotte's. Changes for the better have been made in the food arrangements, the quantity and quality of the food being better, hot night meals with attendance are now set and prepared in the dining-room for night nurses, instead of being taken "where they happen to be on duty, sitting around on cots or bath edges anywhere." Decent crockery is now used in supplying the meals; the pupils are no longer charged with the extra duty when in the wards to attend the Hospital Hall Door as formerly; and Day Nurses are less frequently Called Up From Their Beds In The Night to attend cases. So far so good; but how long will these enforced changes continue, seeing that the hours of many pupil nurses are still outrageously and dangerously so long that no nurse can continue to work, as some of them still do, at Queen Charlotte's, from 1 a.m. to 1 p.m. continuously on duty, and at the end of the twelve full hours proceed to attend classes and lectures for a further period out of the twenty-four on several days in the week? These statements, we are assured, represent the present position of affairs at Queen Charlotte's Hospital.

Having regard to the very large number of women who desire to train as midwives under proper conditions, if the nursing profession is to understand, as the inaction of the authorities of the hospital in question seems to indicate, that the latter are not prepared, nor do they intend to bring their system of training up to modern ideas and requirements, nor to introduce and adhere to definite hours of duty, openly declared and religiously abided by, there would seem to be but one course open. This is to Organise a Great Up-To-Date and Splendidly Equipped Institution for the Training of Pupil Nurses, *where Every Pupil will have full protection and consideration in all respects when pursuing her arduous duties to equip her as a midwife of the highest type.* Of course the authorities of Queen Charlotte's Hospital, in whom the power lies (though we still hope for repentance), may decline to recognise the claims of the women who have trusted this institution and placed themselves unreservedly in its hands in all good faith when joining the school under agreement for training in midwifery.

The time has come for an answer to this question! Is the present inaction of the authorities to be accepted by trained nurses, hospital authorities, in-coming candidates as pupil nurses, and all it may concern, as an intimation that sleep, so far as change and adequate reform are concerned, will continue to be the policy now and in future at Queen Charlotte's Hospital? If so, it may become the duty of the conductors of the chief nursing papers, in justice to the nursing profession and all women who are immediately concerned in this matter, to decline longer to insert the advertisements of Queen Charlotte's Lying-in Hospital, in order to extend adequate warning and protection to an increasing body of women workers, who might suffer as those pupils of this institution have and are suffering, in accordance with the written testimony sent us by the seven signatories and others to prove their case. No one, much less The Hospital, wants to do aught else except to rescue Queen Charlotte's Hospital from the evils which have attached to an old system, carried on without adequate modifications, up-to-date improvements, and the provision throughout to all who enter its doors for training, of everything which will minister to the pupils' comfort, the continuation of sound health, the prevention of unreasonably and dangerously long hours, and other conditions that ought not to exist within the walls of any training-school. The hospital authorities of Queen Charlotte's have still an opportunity to right themselves by making the necessary changes, amending their system and perfecting its efficiency. Failing this, they must be prepared to be superseded by a Splendid Up-to-date Training-School worthy of the British nation and all who take pride in its welfare and progressive development.

